# COPB2: A Novel Prognostic Biomarker That Affects Progression of HCC

**DOI:** 10.1155/2021/6648078

**Published:** 2021-03-20

**Authors:** Jiayao Zhang, Xiaoyu Wang, Guangbing Li, Jingyi He, Ziwen Lu, Yang Yang, Yong Jiang, Liyong Jiang, Feiyu Li, Jun Liu

**Affiliations:** ^1^Department of Hepatobiliary Surgery and Center of Organ Transplantation, Shandong Provincial Hospital, Cheeloo College of Medicine, Shandong University, Jinan, Shandong 250021, China; ^2^Department of Hepatobiliary Surgery and Center of Organ Transplantation, Shandong Provincial Hospital Affiliated to Shandong First Medical University, Jinan, Shandong 250021, China

## Abstract

**Purpose:**

This study is aimed at investigating the expression, underlying biological function, and clinical significance of coatomer protein complex subunit beta 2 (COPB2) in hepatocellular carcinoma (HCC).

**Methods:**

HCC-related data were extracted from The Cancer Genome Atlas (TCGA) database, International Cancer Genome Consortium (ICGC) database, and Gene Expression Omnibus (GEO) database. A logistic regression module was applied to analyze the relationship between the expression of COPB2 and clinicopathologic characteristics. The Cox proportional hazard regression model and Kaplan–Meier method were used for survival analysis. Gene set enrichment analysis (GSEA) was used to annotate the underlying biological functions. Loss-of-function experiments were conducted to determine the underlying mechanisms.

**Results:**

COPB2 was overexpressed in HCC, and high expression of COPB2 was significantly correlated with higher alpha fetoprotein (AFP) (odds ratio (OR) = 1.616, >20 vs. ≤20, *p* < 0.05), stage (OR = 1.744, III vs. I, *p* < 0.05), and grade (OR = 1.746, G4+G3 vs. G2+G1, *p* < 0.05). Kaplan–Meier survival analysis showed that HCC patients with high COPB2 expression had a worse prognosis than those with low COPB2 expression (*p* < 0.0001 for TCGA cohort, *p* < 0.05 for ICGC cohort). The univariate Cox (hazard ratio (HR) = 1.068, *p* < 0.0001) and multivariate Cox (HR = 2.011, *p* < 0.05) regression analyses suggested that COPB2 was an independent risk factor. GSEA showed that mTOR and other tumor-related signaling pathways were differentially enriched in the high COPB2 expression phenotype. Silencing of COPB2 inhibited the proliferation, migration, and invasion abilities by suppressing epithelial-mesenchymal transition and mTOR signaling.

**Conclusion:**

COPB2 is a novel prognostic biomarker and a promising therapeutic target for HCC.

## 1. Introduction

Liver cancer is one of the most common fatal cancers, ranking sixth among cancer diagnoses, and is the fourth leading cause of cancer-related deaths, with 841,000 new cases and 782,000 deaths annually worldwide [1]. The morbidity and mortality of liver cancer rank fourth (10.6%) and third (12.9%), respectively, among all malignant tumors in China [2]. Hepatocellular carcinoma (HCC) accounts for approximately 80% of primary liver cancers; due to its asymptomatic disease progression and limited treatment options, it has become a leading cause of cancer burden globally [1, 3, 4]. There are several ways to treat HCC, such as surgical resection, locoregional therapy, liver transplantation, and systemic therapy; however, its prognosis remains poor, and its survival rate is much lower for patients with metastasis and recurrence [5–8]. Therefore, finding new biomarkers is very important for the prognosis and treatment of HCC and will benefit more patients.

The coatomer protein complex subunit beta 2 (COPB2), encoded by a gene located on chromosome 3q23 [9], is one of the seven subunits that form coatomer complex I (COPI), which is one of the three types of coat proteins (COPs) that play a key role in intracellular transport by forming transport vesicles [10]. Previous studies have reported that the main functions of COPB2 are the regulation of extracellular membrane transport and mediation of retrograde transport from the Golgi complex to the endoplasmic reticulum (ER) [11–13]. Recently, COPB2 was reported to have important correlations with various cancer types and has different functions in different tumors, such as breast cancer, glioma, and prostate cancer [14–16]. Silencing COPB2 can inhibit the proliferation of colon cancer cells by inducing cell cycle arrest [17]. In addition, coatomer protein complex subunit alpha (COPA), another subunit of COPI, is an important paralog of COPB2 [18] and has been reported to be upregulated in tumors relative to paired adjacent nonmalignant tissues in patients with liver cancer [19]. It was also reported that reduced editing of *COPA* was implicated in the pathogenesis of HCC and editing of *COPA^WT^* may switch it from a tumor-promoting gene to a tumor suppressor by deactivating the PI3K/AKT/mTOR pathway through downregulation of caveolin-1 (CAV1) [20]. Deregulated mTOR signaling significantly contributes to the molecular pathogenesis of HCC [21]. Considering the relationship between COPB2 and other tumors and the role of its paralog, COPA, in HCC, we hypothesized that COPB2 may play an important role in the progression of HCC and may be a new potential therapeutic target for HCC. By querying the online UALCAN database (http://ualcan.path.uab.edu/analysis.html), we found that COPB2 expression was elevated in HCC and correlated with its prognosis [22], but no research has yet revealed the mechanism by which COPB2 regulates the malignant progression of HCC.

In this study, we explored the role of COPB2 in HCC by analyzing HCC-related data from The Cancer Genome Atlas (TCGA) database, the International Cancer Genome Consortium (ICGC) database, and Gene Expression Omnibus (GEO) databases, as well as conducting a series of experiments. The results of the current study revealed that COPB2 is a novel prognostic biomarker and a promising therapeutic target for HCC.

## 2. Material and Methods

### 2.1. Bioinformatics Analysis

All HCC-related data (including clinical information and corresponding mRNA expression data) were downloaded from The Cancer Genome Atlas (TCGA) database (https://portal.gdc.cancer.gov/repository) and the International Cancer Genome Consortium (ICGC) database (https://dcc.icgc.org/releases). We then used Perl (v 5.26.3) and R (v 3.6.3) to sort and extract the data and merged the expression data with clinical data. Cases without clinical data or expression data were excluded. At the same time, patients with a pathological type other than primary hepatocellular carcinoma were also excluded; 370 HCC cases from TCGA cohort and 232 HCC cases from the ICGC cohort were used for subsequent analysis. The patients' basic information is shown in Tables S1 and S2. The clinical characteristics of TCGA cohort included age, sex, body mass index (BMI), T stage, lymph node (N), metastasis (M), stage, grade, tumor status, family history of cancer, vascular invasion, AFP, new tumor event, survival status, and survival time, while the ICGC cohort included age, sex, stage, grade, and tumor status. Cases with incomplete clinical pathological information were included in the analysis based on the available clinical information and excluded from the analysis of the clinical pathological features where data were missing. In the survival analysis, patients with a survival time of less than 30 days were excluded, since they may have died of serious complications (including bleeding, intracranial infections, and heart failure) rather than HCC. To further verify the expression level of COPB2 mRNA in patients with HCC, six datasets from the Gene Expression Omnibus (GEO) database were used (Table S3). In the present study, in addition to difference analysis and Kaplan–Meier analysis, the logistic regression and Cox proportional hazard regression models were used for clinical correlation analysis and survival analysis, respectively. Gene set enrichment analysis (GSEA) is a method to identify classes of genes or proteins that are overrepresented in a large set of genes or proteins and may be associated with disease phenotypes [23]. GSEA was used to explore the potential biological signaling pathways related to COPB2 in HCC. During each analysis, all genes were generated in an ordered list and were classified into high and low COPB2 expression phenotypes. Gene set permutations were performed 1000 times. A nominal *p* value < 0.05 and false discovery rate (FDR) < 0.05 were used to filter the pathways enriched in each phenotype.

### 2.2. Cell Culture and siRNA Transfection

BEL7402 and SMMC7721 HCC cell lines were purchased from the BeNa Culture Collection (Beijing, China). All cells were cultured in RPMI 1640 medium supplemented with 10% fetal bovine serum (FBS) in a humidified chamber with 5% CO_2_ at 37°C. siRNA for COPB2 was purchased from Genomeditech (Shanghai, China). BEL7402 and SMMC7721 cells were seeded in six-well plates at 30–50% confluence and were then transfected with 50 nmol/L siRNAs using Lipofectamine 3000 reagent (Thermo, L3000015, Waltham, MA, USA). The cells were collected following transfection efficiency determination and follow-up experiments after being transfected for 48–72 hours.

### 2.3. CCK-8 Assay

The transfected BEL7402 and SMMC7721 cells were seeded and cultured in four 96-well plates at 2000 cells/well, with five replicate wells for each group; the cell viability of each group was measured after 0 h, 24 h, 48 h, and 72 h at a wavelength of 450 nm with a microplate reader after adding CCK-8 reagent for 3 h in each well.

### 2.4. Immunohistochemistry

Tumor tissues and corresponding adjacent nontumor tissues in 20 HCC patients undergoing hepatectomy were fixed with 4% paraformaldehyde immediately after isolation and then embedded in paraffin for being cut into 5 *μ*m thick continuous sections. These sections were then deparaffinized, hydrated, and incubated overnight with the primary rabbit anti-COPB2 polyclonal antibody (Abcam, ab192924, CA, USA) and primary rabbit anti-phospho-mTOR (Ser2448) polyclonal antibody (CST, 2796, MA, USA) overnight at 4°C. On the next day, the cells were incubated with the secondary antibody at 37°C and then visualized using a DAB kit (ZSGB-BIO, ZLI-9017, Beijing, China) and counterstained with hematoxylin. The expression level was independently evaluated by two senior pathologists using the H-score method.

### 2.5. Scratch Wound Healing Assay

The transfected BEL7402 and SMMC7721 cells were seeded in six-well plates. When they reached approximately 80–90% confluence, the cells were scratched using a 200 *μ*L pipette tip. Serum-free medium was added after washing with phosphate-buffered saline to remove debris. Photographs were taken at 0 h and 48 h to compare wound healing rates.

### 2.6. Transwell Assay

For the migration assay, 4 × 10^4^ cells (200 *μ*L serum-free cell suspension) were seeded into the upper Transwell chamber with 8 *μ*m pore inserts (Corning, NY, USA), while the bottom chamber was filled with 600 *μ*L RPMI 1640 medium supplemented with 10% fetal bovine serum. After incubation at 37°C with 5% CO_2_ for 24 h, cells invading the lower surfaces were fixed with 4% paraformaldehyde and stained with 0.1% crystal violet stain solution, while cells on the upper surface were scraped. Nine random fields were used for statistical analysis. For the invasion assay, 1 × 10^5^ cells (200 *μ*L serum-free cell suspension) were seeded into the upper Transwell chamber, which was prepaved with Matrigel. The remaining steps were the same as those for the migration assay.

### 2.7. Cell Cycle Distribution

All cells were collected, fixed, and stained after being transfected for 72 h, and the cell cycle distribution was detected using a Muse Cell Analyzer (Merck & Millipore, Germany). All experimental procedures were performed in accordance with the manufacturer's protocol.

### 2.8. Western Blotting

The total protein in each group of cells was lysed in PIPA lysis buffer (Solarbio, R0010) supplemented with phenylmethylsulfonyl fluoride (PMSF) protease inhibitor (Thermo Scientific, 36987, Waltham, MA, USA) and phosphatase inhibitor (Thermo Scientific, 78428, Waltham, MA, USA); and their concentrations were then measured using a BCA Protein Assay Kit (Solarbio, PC0020, Beijing, China). 30 *μ*g/well of protein extracts was separated on 10% SDS-PAGE and transferred onto Nitrocellulose Transfer Membrane (PALL, 66485, NY, USA). After cutting into different strips according to the molecular weight of target proteins, the membranes were reacted with primary antibodies against target proteins overnight on a shaker at 4°C. On the next day, these bands were visualized after incubation with the secondary antibody.

### 2.9. Statistical Analysis

GraphPad Prism 8.0, SPSS 25.0, and R 3.6.3 software were used for all statistical analyses. The distribution of all data was tested for normality prior to statistical analysis. When comparing the differences between two groups, we used the *t*-test for normally distributed data and used a nonparametric test (unpaired: Mann–Whitney *U* test; paired: Wilcoxon matched-pairs signed rank-test) for the data that were not normally distributed. The data of CCK-8 assays was analyzed using two-way repeated measurement ANOVA with Sidak's multiple comparisons test. Survival was analyzed using a Kaplan–Meier plot and log-rank test. The correlation analysis between COPB2 expression level and clinicopathological parameters in HCC patients used logistic regression. The correlation between different clinicopathological variables and overall survival was explored using univariate and multivariate Cox proportional hazard regression mode. *p* < 0.05 was considered to be a significant statistical difference.

## 3. Results

### 3.1. COPB2 Overexpressed in HCC

**Figure 1:**
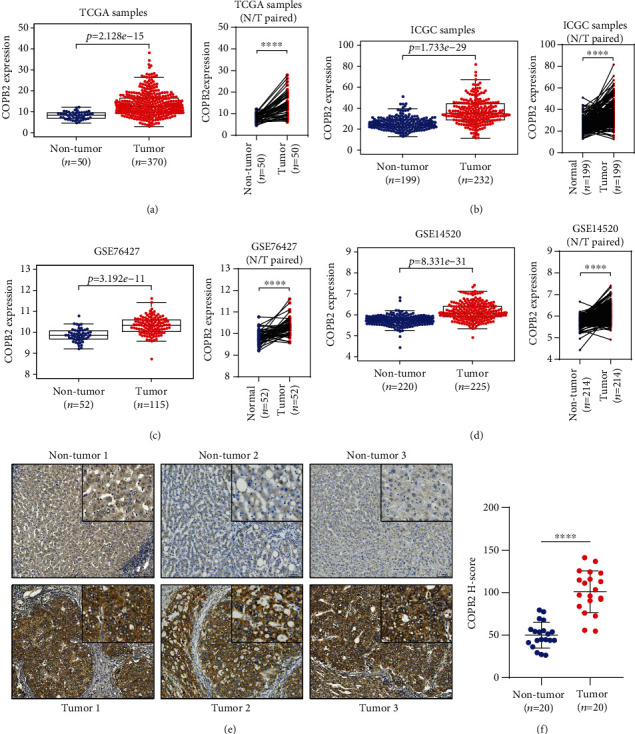
COPB2 overexpressed in HCC. (a) Comparison of COPB2 mRNA expression in tumor (*n* = 370) and nontumor tissues (*n* = 50) in patients with HCC from TCGA database using paired (Wilcoxon matched-pairs signed rank test, *p* < 0.0001) and unpaired (Mann–Whitney *U* test, *p* < 0.0001) analyses. (b) Comparison of COPB2 mRNA expression in tumor (*n* = 232) and nontumor (*n* = 199) tissues in patients with HCC from ICGC database using paired (Wilcoxon matched-pairs signed rank test, *p* < 0.0001) and unpaired (Mann–Whitney *U* test, *p* < 0.0001) analyses. (c) Comparison of COPB2 mRNA expression in tumor (*n* = 115) and nontumor tissues (*n* = 52) in patients with HCC from the GSE76472 dataset using paired (Wilcoxon matched-pairs signed rank test, *p* < 0.0001) and unpaired (Mann–Whitney *U* test, *p* < 0.0001) analyses. (d) Comparison of COPB2 mRNA expression in tumor (*n* = 225) and nontumor tissues (*n* = 220) in patients with HCC from the GSE76472 dataset using paired (Wilcoxon matched-pairs signed rank test, *p* < 0.0001) and unpaired (Mann–Whitney *U* test, *p* < 0.0001) analyses. (e, f) Immunohistochemical analysis of COPB2 in HCC tissues (*n* = 20) and adjacent nontumor tissues (*n* = 20) (*t*-test, *p* < 0.0001). ^∗^*p* < 0.05, ^∗∗^*p* < 0.01, ^∗∗∗^*p* < 0.001, and ^∗∗∗∗^*p* < 0.0001.

The mRNA expression data of 370 HCC tissues and 50 matched nontumor tissues from TCGA cohort were analyzed. The results showed that COPB2 mRNA was significantly overexpressed in HCC tissues compared with the expression in nontumor tissues using unpaired and paired tests (Figure 1(a); unpaired: *p* < 0.0001, paired: *p* < 0.0001). For the ICGC cohort, 232 HCC tissues and 199 matched nontumor tissues were analyzed, and the results were consistent with those of TCGA cohort (Figure 1(b); unpaired: *p* < 0.0001, paired: *p* < 0.0001). To further verify the expression level of COPB2 mRNA in patients with HCC, six datasets from the GEO database were analyzed and similar results were obtained: GES76427 (tumor = 115, nontumor = 52) (Figure 1(c); unpaired: *p* < 0.0001, paired: *p* < 0.0001), GSE14520 (tumor = 225, nontumor = 220) (Figure 1(d); unpaired: *p* < 0.0001, paired: *p* < 0.0001), GSE39791 (tumor = 72, nontumor = 72) (Figure S1A; unpaired: *p* < 0.0001, paired: *p* < 0.0001), GES36411 (tumor = 42, nontumor = 42) (Figure S1B; unpaired: *p* < 0.001), GSE102079 (tumor = 152, nontumor = 105) (Figure S1C; unpaired: *p* < 0.01), and GSE25097 (tumor = 268, nontumor = 289) (Figure S1D; unpaired: *p* < 0.001). In order to verify the results of the above bioinformatics analysis, we performed immunohistochemical staining on tumor tissues (*n* = 20) and matched nontumor tissues (*n* = 20) from HCC patients; as expected, the results showed that COPB2 was significantly overexpressed in tumor tissues (Figures 1(e) and 1(f), *p* < 0.0001).

### 3.2. High COPB2 Expression Was Correlated with Poor Prognosis in HCC Patients

**Figure 2:**
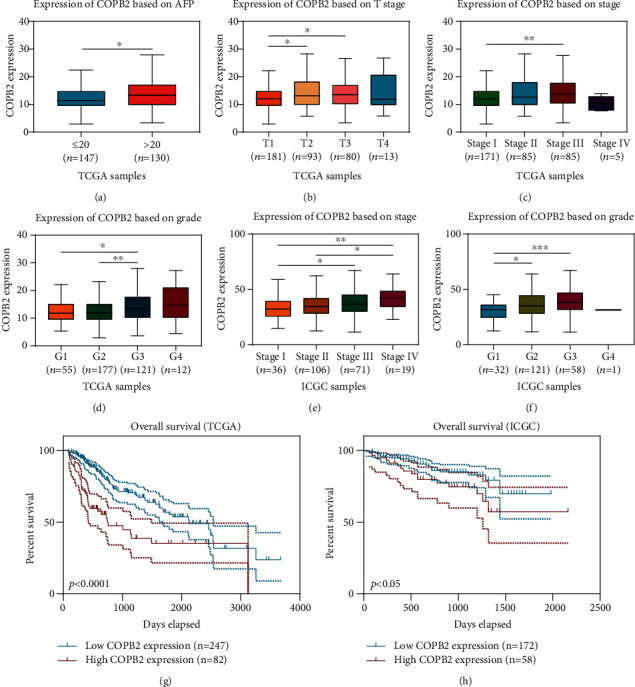
High COPB2 expression was correlated with poor prognosis in HCC patients. (a) Expression of COPB2 based on AFP in patients with HCC from TCGA cohort: AFP ≤ 20 (*n* = 147) vs. AFP > 20 (*n* = 130), *p* < 0.05 (Mann–Whitney *U* test). (b) Expression of COPB2 based on T stage in patients with HCC from TCGA cohort: T3 (*n* = 80) vs. T1 (*n* = 181), *p* < 0.05; T2 (*n* = 93) vs. T1 (*n* = 181), *p* < 0.05 (Mann–Whitney *U* test). (c) Expression of COPB2 based on stage in patients with HCC from TCGA cohort: Stage III (*n* = 85) vs. Stage I (*n* = 171), *p* < 0.01 (Mann–Whitney *U* test). (d) Expression of COPB2 based on grade in patients with HCC from TCGA cohort: G3 (*n* = 121) vs. G1 (*n* = 55), *p* < 0.05; G3 (*n* = 121) vs. G2 (*n* = 177), *p* < 0.01 (Mann–Whitney *U* test). (e) Expression of COPB2 based on stage in patients with HCC from the ICGC cohort: Stage IV (*n* = 19) vs. Stage I (*n* = 36), *p* < 0.01; Stage IV (*n* = 19) vs. Stage II (*n* = 106), *p* < 0.05; Stage III (*n* = 71) vs. Stage I (*n* = 36), *p* < 0.05 (Mann–Whitney *U* test). (f) Expression of COPB2 based on grade in patients with HCC from the ICGC cohort: G3 (*n* = 58) vs. G1 (*n* = 32), *p* < 0.001; G2 (*n* = 121) vs. G1 (*n* = 32), *p* < 0.05 (Mann–Whitney *U* test). (g, h) Effect of COPB2 expression level on overall survival of HCC patients in TCGA (*p* < 0.0001) and ICGC (*p* < 0.05) cohorts (cutoff: upper quartile) (Kaplan–Meier plot and log-rank test). ^∗^*p* < 0.05, ^∗∗^*p* < 0.01, ^∗∗∗^*p* < 0.001, and ^∗∗∗∗^*p* < 0.0001.

We conducted a further correlation analysis on the expression data and clinical data of HCC cases from TCGA and ICGC databases. The results indicated that high expression levels of COPB2 positively correlated with the clinical characteristics of poor prognosis. There were significant differences in COPB2 expression between different subgroups defined based on AFP (≤20 (*n* = 147) vs. >20 (*n* = 130), *p* < 0.05), T stage (T2 (*n* = 93) vs. T1 (*n* = 181), *p* < 0.05; T3 (*n* = 80) vs. T1 (*n* = 181), *p* < 0.05), stage (Stage III (*n* = 85) vs. Stage I (*n* = 171), *p* < 0.01), and grade (G3 (*n* = 121) vs. G1 (*n* = 55), *p* < 0.05; G3 (*n* = 121) vs. G2 (*n* = 177), *p* < 0.01) in TCGA cohort (Figures 2(a)–2(d)) and stage (Stage IV (*n* = 19) vs. Stage I (*n* = 36), *p* < 0.01; Stage IV (*n* = 19) vs. Stage II (*n* = 106), *p* < 0.05; Stage III (*n* = 71) vs. Stage I (*n* = 36), *p* < 0.05) and grade (G3 (*n* = 58) vs. G1 (*n* = 32), *p* < 0.001; G2 (*n* = 121) vs. G1 (*n* = 32), *p* < 0.05) in the ICGC cohort (Figures 2(e) and 2(f)). Meanwhile, a logistic regression analysis of TCGA cohort also revealed similar results (Table 1).

Kaplan–Meier survival analysis (cases with a survival time of less than 30 days were not considered) indicated that HCC patients with high COPB2 expression had a more unfavorable prognosis than those with low COPB2 expression in both TCGA (Figure 2(g), high (*n* = 82) vs. low (*n* = 247), *p* < 0.0001) and ICGC cohorts (Figure 2(h), high (*n* = 58) vs. low (*n* = 172), *p* < 0.05). The upper quartile value of COPB2 expression levels was used as the cutoff point [22].

Univariate and multivariate Cox proportional hazard regression analyses were performed on TCGA cohort. In the univariate Cox analysis, shorter overall survival (OS) was found in those with higher expression of COPB2 (hazard ratio (HR) = 1.068, 95% confidence interval (CI): 1.037−1.099, *p* < 0.0001), higher T stage (HR = 1.665, 95% CI: 1.390−1.993, *p* < 0.0001), worse pathological stage (HR = 1.652, 95% CI: 1.349−2.024, *p* < 0.0001), and “with tumor” status (HR = 1.604, 95% CI: 1.116−2.306, *p* < 0.05) (Table 2). However, in the multivariate Cox analysis, worse OS was only significantly associated with high expression of COPB2 (HR = 2.011, 95% CI: 1.111−3.641, *p* < 0.05) (Table 2). This indicates that COPB2 was an independent prognostic factor for HCC.

In summary, the above results indicated that high COPB2 expression correlated with poor prognosis in HCC.

### 3.3. GSEA Identified COPB2-Related Biological Signaling Pathways in HCC

**Figure 3:**
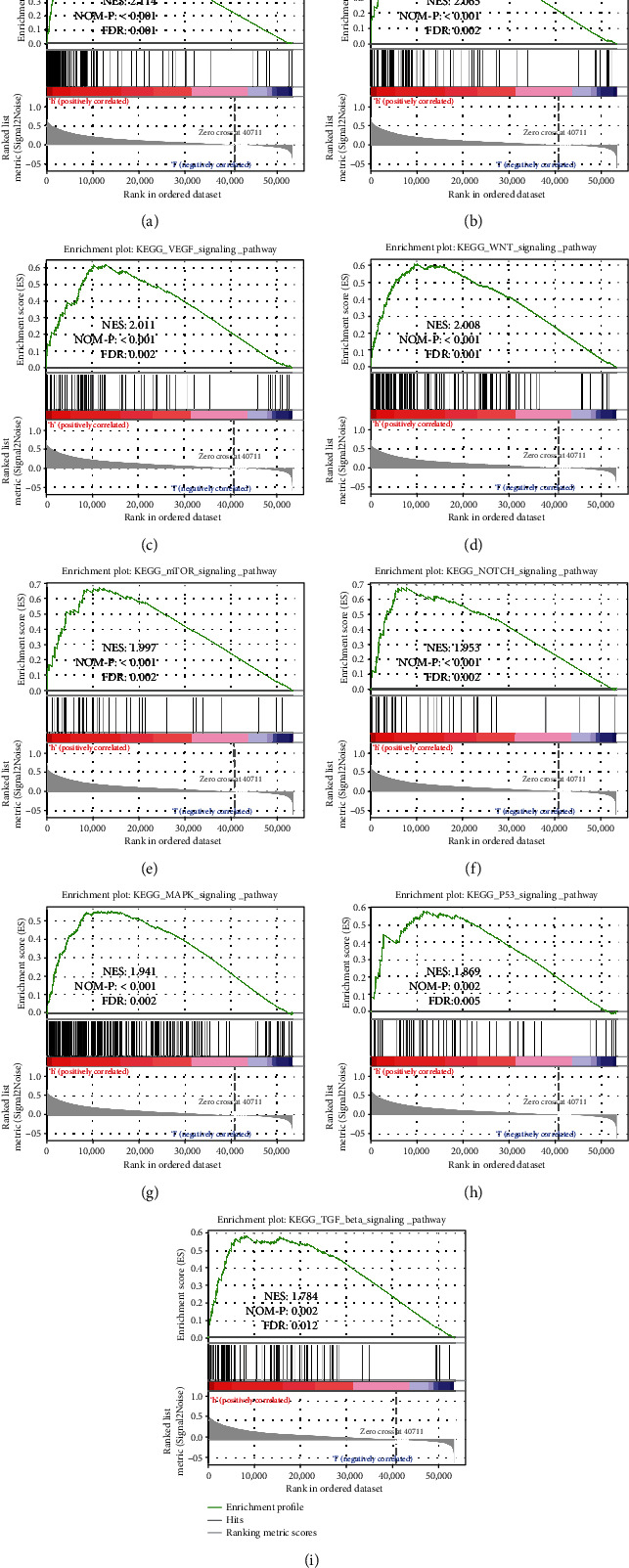
GSEA identified COPB2-related biological signaling pathways in HCC. (a) Cell cycle. (b) ERBB signaling pathway. (c) VRGF signaling pathway. (d) WNT signaling pathway. (e) mTOR signaling pathway. (f) NOTCH signaling pathway. (g) MAPK signaling pathway. (h) P53 signaling pathway. (i) TGF-*β* signaling pathway. ES: enrichment score; NES: normalized ES; NOM-P: normalized *p* value; FDR: false discovery rate.

To explore the biological signaling pathways involved in COPB2 expression in HCC, we performed GSEA of the high and low COPB2 expression groups in TCGA cohort. The results revealed a great number of significant differences (false discovery rate (FDR) < 0.05, *p* < 0.05) in the enrichment of the Molecular Signatures Database (MSigDB) Collection (c2.cp.kegg.v7.2.symbols.gmt), and we observed that the cell cycle (Figure 3(a), normalized enrichment score (NES): 2.114, FDR: 0.001, *p* < 0.001), ERBB signaling pathway (Figure 3(b), NES: 2.065, FDR: 0.002, *p* < 0.001), VEGF signaling pathway (Figure 3(c), NES: 2.011, FDR: 0.002, *p* < 0.001), WNT signaling pathway (Figure 3(d), NES: 2.008, FDR: 0.001, *p* < 0.001), mTOR signaling pathway (Figure 3(e), NES: 1.997, FDR: 0.002, *p* < 0.001), NOTCH signaling pathway (Figure 3(f), NES: 1.953, FDR: 0.002, *p* < 0.001), MAPK signaling pathway (Figure 3(g), NES: 1.941, FDR: 0.002, *p* < 0.001), P53 signaling pathway (Figure 3(h), NES: 1.869, FDR: 0.005, *p* < 0.01), and TGF-*β* signaling pathway (Figure 3(i), NES: 1.784, FDR: 0.012, *p* < 0.01) were differentially enriched in those with the high COPB2 mRNA expression phenotype. The results indicated that COPB2 may play a vital role in the occurrence and progression of HCC.

### 3.4. Knockdown of COPB2 Suppressed Migration and Invasion of HCC Cell Lines

**Figure 4:**
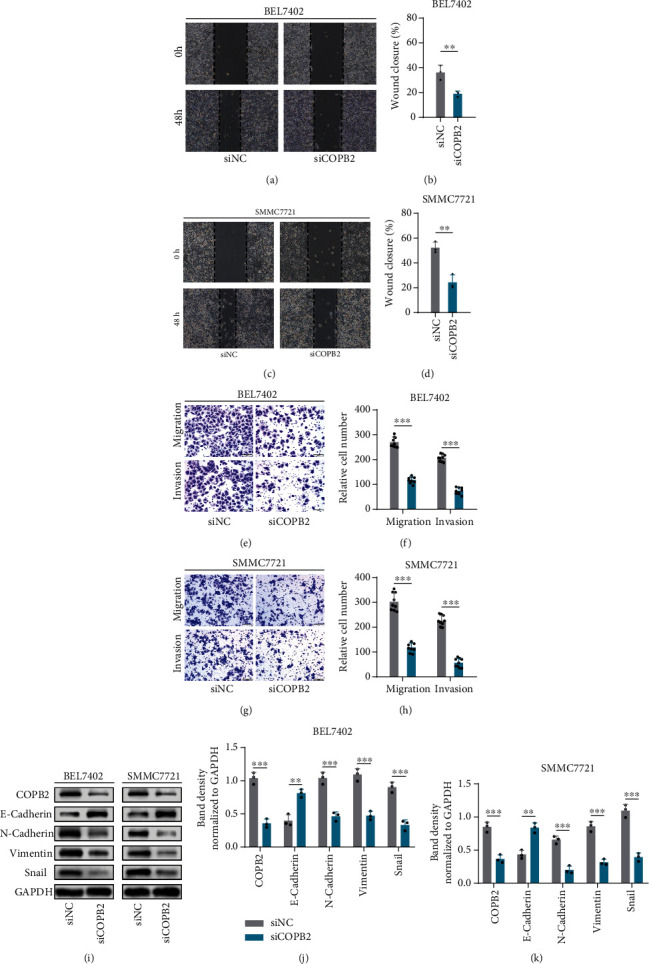
Knockdown of COPB2 suppressed migration and invasion in HCC cell lines. (a, c) Wound healing assays detected the cell migration ability of BEL7402 and SMMC7721 cells transfected with siNC or siCOPB2; the representative images were obtained at different time points. (b, d) Statistical analysis of the results of wound healing assays (*n* = 3). (e, g) Transwell assays were used to detect the cell migration and invasion ability of BEL7402 and SMMC7721 cells transfected with siNC and siCOPB2; the representative images are displayed. (f, h) Statistical analysis of the results of the Transwell assays (*n* = 9). (i) Representative images of western blotting analysis of COPB2, E-cadherin, N-cadherin, vimentin, Snail, and GAPDH in BEL7402 and SMMC7721 cells transfected with siNC and siCOPB2. GAPDH was used as the loading control. (j, k) Statistical analysis of gray values of western blotting assays (*n* = 3). All data were analyzed using the *t*-test and are displayed as mean ± standard deviation (SD). ^∗^*p* < 0.05, ^∗∗^*p* < 0.01, ^∗∗∗^*p* < 0.001, and ^∗∗∗∗^*p* < 0.0001.

GSEA results showed that overexpression of COPB2 positively correlated with the activation of many tumor-related pathways in HCC. To verify the results of GSEA, we performed a series of experiments at the cellular level. All experiments were repeated at least three times. Wound healing assays showed that the migration ability of the COPB2 knockdown group was significantly weaker than that of the vector-transfected control group in both the BEL7402 (Figures 4(a) and 4(b), *p* < 0.01) and SMMC7721 (Figures 4(c) and 4(d), *p* < 0.01) cell lines. Transwell assays verified that downregulation of COPB2 significantly inhibited the migration (BEL7402, *p* < 0.001; SMMC7721, *p* < 0.001) and invasion (BEL7402, *p* < 0.001; SMMC7721, *p* < 0.001) abilities of both cell lines (Figures 4(e)–4(h)). Moreover, we also measured the change in epithelial-mesenchymal transition- (EMT-) related protein expression levels using western blotting assays. The results revealed that the protein level of E-cadherin was markedly elevated (BEL7402, *p* < 0.01; SMMC7721, *p* < 0.01), while the expression of N-cadherin (BEL7402, *p* < 0.001; SMMC7721, *p* < 0.001), vimentin (BEL7402, *p* < 0.001; SMMC7721, *p* < 0.001), and Snail (BEL7402, *p* < 0.001; SMMC7721, *p* < 0.001) was significantly downregulated in both cell lines after COPB2 knockdown (Figures 4(i)–4(k)).

### 3.5. Silencing of COPB2 Inhibits the Proliferation by Inhibiting mTOR Signaling

**Figure 5:**
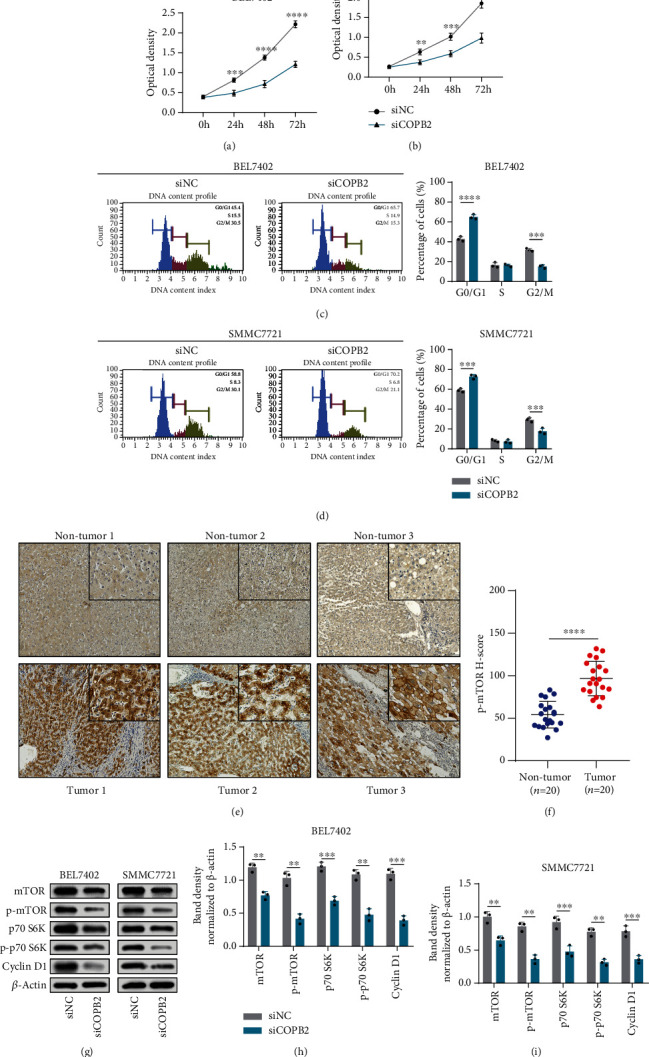
Silencing of COPB2 inhibits the proliferation by inhibiting mTOR signaling. (a, b) BEL7402 and SMMC7721 cells were transfected with siNC and siCOPB2, respectively, and cell viability was analyzed using the CCK-8 assays (*n* = 5) (two-way repeated measurement ANOVA with Sidak's multiple comparisons test). (c, d) Detection of the cell cycle distribution of BEL7402 and SMMC7721 cells after being transfected with siNC and siCOPB2 (*n* = 3) (*t*-test). (e, f) Immunohistochemical analysis of p-mTOR in HCC tissues (*n* = 20) and adjacent nontumor tissues (*n* = 20) (*t*-test). (g) Representative images of western blotting analysis of mTOR, p-mTOR, p70 S6K, p-p70 S6K, cyclin D1, and *β*-actin in BEL7402 and SMMC7721 cells transfected with siNC and siCOPB2. #x03B2;-actin was used as the loading control. (h, i) Statistical analysis of gray values of western blotting assays (*n* = 3) (*t*-test). All data are displayed as mean ± standard deviation (SD). ^∗^*p* < 0.05, ^∗∗^*p* < 0.01, ^∗∗∗^*p* < 0.001, and ^∗∗∗∗^*p* < 0.0001.

In order to explore whether COPB2 can affect the proliferation of HCC, we performed CCK-8 assays. As expected, cells transfected with siCOPB2 had a lower rate of proliferation than siNC-treated cells in both BEL7402 (Figure 5(a); 24 h: *p* < 0.01, 48 h: *p* < 0.0001, 72 h: *p* < 0.0001) and SMMC7721 (Figure 5(b); 24 h: *p* < 0.01, 48 h: *p* < 0.001, 72 h: *p* < 0.0001) cell lines. In addition, we examined their cell cycle distribution and observed that compared with the siNC group, there was a significant increase in the number of cells in the G0/G1 phase and a decrease in the number of cells in the G2/M phase in the siCOPB2 group in both the BEL7402 (Figure 5(c), G0/G1: *p* < 0.001; G2/M: *p* < 0.001) and SMMC7721 (Figure 5(d), G0/G1: *p* < 0.001; G2/M: *p* < 0.001) cell lines. GSEA results suggested that the activation of the mTOR signaling pathway was closely associated with overexpression of COPB2 in HCC. To further confirm this, we performed immunohistochemical staining on tumor tissues and matched nontumor tissues in HCC patients. As expected, the results show that phospho-mTOR was significantly overexpressed in tumor tissues (Figures 5(e) and 5(f), *p* < 0.0001). In addition, the activity of this pathway of HCC cell lines was examined using a western blotting assay. In the present study, we observed that after knocking down COPB2, the expression level of mTOR (BEL7402, *p* < 0.01; SMMC7721, *p* < 0.01) and p70 S6K (BEL7402, *p* < 0.001; SMMC7721, *p* < 0.001) as well as phospho-mTOR (BEL7402, *p* < 0.01; SMMC7721, *p* < 0.01), phospho-p70 S6K (BEL7402, *p* < 0.01; SMMC7721, *p* < 0.01), and their downstream protein cyclin D1 (BEL7402, *p* < 0.001; SMMC7721, *p* < 0.001) decreased in both cell lines (Figures 5(g)–5(i)). These results suggest that silencing of COPB2 inhibits cell proliferation and that the mTOR signaling pathway plays an important role.

## 4. Discussion

HCC accounts for approximately 80% of primary liver cancers [1]. Due to its asymptomatic disease progression and lack of effective methods to make an early diagnosis, HCC is often diagnosed at an advanced stage [6]; its typically late-stage presentation, limited treatment options, and aggressive nature lead to it having a very poor prognosis [4, 24, 25]. In China, digestive tract cancers account for 36.4% of cancer-related deaths, of which liver cancer account for more than one-third [2]. Therefore, there is an urgent need to identify effective biomarkers for the diagnosis and prognosis of HCC, as well as therapeutic targets.

COPB2 is a 102 kDa protein that was first identified in 1993 [26, 27]. Previous research confirmed that COPB2 is an element of non-clathrin-coated vesicles and is involved in regulating membrane transport in extracellular pathways [9, 28]. In addition, as a subunit of the Golgi coatomer complex, COPB2 is essential for retrograde transport from the Golgi complex to the endoplasmic reticulum [11–13]. Compared with normal cells, the biosynthetic activity of tumor cells is abnormally vigorous [29]. As is well known, the Golgi complex plays an important role in anabolism; thus, COPB2 is certain to play a very important role in the occurrence and progression of tumors. Recently, the functions of COPB2 in tumors have been increasingly studied. In gliomas, COPB2 has been reported to be an important factor in the regulation of the immune microenvironment, and its high expression is related to adverse outcomes [14]. In breast cancer, COPB2 may predict metastasis [15]. In gastric cancer, COPB2 can affect the growth and apoptosis of gastric cancer cell lines via the RTK signaling pathway [30]. In lung adenocarcinoma, COPB2 was confirmed to be overexpressed and negatively correlated with survival, and COPB2 downregulation enhanced apoptosis and repressed proliferation and tumorigenesis in lung adenocarcinoma cells [31]. In prostate cancer, COPB2 has also been shown to be highly expressed and can promote PC-3 cell proliferation and inhibit apoptosis by affecting its cell cycle [16]. Downregulation of COPB2 could inhibit the growth of human cholangiocellular carcinoma cells [32]. It has also been reported that reduced editing of *COPA*, an important paralog of COPB2, has been implicated in the pathogenesis of HCC, and editing of *COPA*^WT^ may switch it from a tumor-promoting gene to a tumor suppressor by deactivating the PI3K/AKT/mTOR pathway through downregulation of caveolin-1 (CAV1) [20]. These findings suggest an essential role of COPB2 in the occurrence and progression of tumors, which provides a good theoretical basis for our study of the role of COPB2 in HCC.

With the advancement of technology, high-throughput sequencing technology has been increasingly used in cancer research [33, 34]. In the present study, we explored the role of COPB2 in human HCC and the underlying mechanism using database analysis combined with basic experiments. Bioinformatic analysis based on TCGA, ICGC, and GEO databases revealed that COPB2 mRNA levels were higher in HCC tissues than in nontumor tissues. At the same time, we confirmed the high expression of COPB2 protein in HCC tissues using immunohistochemical assay. The mRNA expression data and clinical information of HCC were then analyzed. Correlation and survival analyses showed that high COPB2 expression was closely correlated with advanced clinicopathological parameters (higher AFP, worse T stage, poor pathological stage, and higher grade) and worse prognosis. Univariate and multivariate Cox analyses indicated that COPB2 was an independent prognostic factor for HCC. GSEA suggested that various signaling pathways closely related to tumor occurrence and development [35–39] (e.g., mTOR signaling pathway, WNT signaling pathway, VEGF signaling pathway, and NOTCH signaling pathway) were differentially enriched in those with the high COPB2 expression phenotype. To further explore the role of COPB2 in HCC, we performed a series of experiments. Functional investigations indicated that downregulation of COPB2 significantly inhibited the proliferation, migration, and invasion capacity of HCC in vitro. In addition, mechanistic experiments demonstrated that deletion of COPB2 significantly restrained EMT and activation of the mTOR signaling pathway.

The occurrence and progression of tumors are associated with abnormal regulation of multiple signaling pathways. EMT plays a vital role in tumorigenesis and tumor progression and is closely related to tumor invasion and migration abilities [40, 41]. Common signaling pathways, such as the WNT, NOTCH, MAPK, and TGF-*β* signaling pathways, can activate EMT regulators [39, 42–44]. Evidence indicates that the mTOR signaling pathway governs cell growth and is activated in cancer [35, 45]. The GSEA results showed that these signaling pathways were all enriched in the high COPB2 expression group in HCC, and the results of function and mechanism experiments are also consistent with this.

The results of the current study showed that COPB2 is overexpressed in HCC tissues, associated with HCC prognosis, and plays a crucial role in the proliferation, migration, and invasion of HCC cell lines in vitro, indicating that COPB2 is a novel prognostic biomarker and promising therapeutic target for HCC.

This study has some limitations. First, the tumor tissue specimens of the patients were usually obtained during surgery; however, patients with distant metastasis generally do not have indications for surgery. Consequently, the expression data from this patient population are rarely obtained. Second, according to the results of the GSEA, COPB2 may also influence the progression of HCC through other signaling pathways other than the mTOR signaling pathway; however, the current research on the relationship between COPB2 and HCC is in its infancy, and a lot of work is needed to explore whether COPB2 can affect HCC through other pathways in subsequent studies. Finally, this study only included cases from two cohorts, and a multicenter study should be conducted in the future.

## 5. Conclusion

COPB2 is a novel prognostic biomarker and a promising therapeutic target of HCC.

## Figures and Tables

**Table 1:** Correlations between COPB2 mRNA expression and clinicopathological characteristics (logistic regression).

Clinical characteristics	Total (*N*)	OR in COPB2 expression	*p* value
Age (continuous)	370	0.986 (0.971−1.001)	0.067
Sex (male vs. female)	370	0.726 (0.468−1.122)	0.150
Status (with tumor vs. tumor-free)	343	1.154 (0.732−1.821)	0.538
Vascular invasion (positive vs. negative)	314	1.000 (0.627−1.594)	1.000
AFP (>20 vs. ≤20)	277	1.616 (1.006−2.606)	0.048^∗^
T stage (T3+T2 vs. T1)	354	1.539 (1.013−2.345)	0.044^∗^
Stage (III vs. I)	256	1.744 (1.033−2.969)	0.038^∗^
Grade (G4+G3 vs. G2+G1)	365	1.746 (1.136−2.695)	0.011^∗^

OR: odds ratio. ^∗^*p* < 0.05.

**Table 2:** Univariate and multivariate analyses of the correlation of COPB2 with OS among HCC patients from TCGA cohort.

Clinical characteristics	HR	*p* value
Univariate analysis		
Age (continuous)	1.250 (0.882−1.772)	0.210
Sex (male vs. female)	0.805 (0.564−1.147)	0.230
BMI (continuous)	0.974 (0.941−1.007)	0.124
T stage (T4/T3/T2/T1)	1.665 (1.390−1.993)	<0.0001^∗∗∗∗^
Lymph nodes (positive vs. negative)	1.948 (0.477−7.952)	0.353
Distant metastasis (positive vs. negative)	3.820 (1.201−12.146)	0.023^∗^
Stage (IV/III/II/I)	1.652 (1.349−2.024)	<0.0001^∗∗∗∗^
Grade (G4/G3/G2/G1)	1.127 (0.892−1.424)	0.317
Tumor status (with tumor vs. tumor-free)	1.604 (1.116−2.306)	0.011^∗^
Family cancer history (yes vs. no)	1.182 (0.819−1.707)	0.372
New tumor event (yes vs. no)	1.335 (0.932−1.913)	0.116
COPB2 expression (continuous)	1.068 (1.037−1.099)	<0.0001^∗∗∗∗^
Multivariate analysis		
T stage (T4/T3/T2/T1)	2.074 (0.816−5.270)	0.125
Distant metastasis (positive vs. negative)	1.642 (0.282−9.544)	0.581
Stage (IV/III/II/I)	0.772 (0.269−2.215)	0.630
Tumor status (with tumor vs. tumor-free)	1.002 (0.487−2.064)	0.995
COPB2 expression (continuous)	2.011 (1.111−3.641)	0.021^∗^

HR: hazard ratio. ^∗^*p* < 0.05 and ^∗∗∗∗^*p* < 0.0001.

## Data Availability

The data used to support this study are available from the corresponding author upon request.

## Abbreviations

HCC: Hepatocellular carcinoma
COPB2: Coatomer protein complex subunit beta 2
TCGA: The Cancer Genome Atlas
ICGC: International Cancer Genome Consortium
GEO: Gene Expression Omnibus
GSEA: Gene set enrichment analysis
AFP: Alpha fetoprotein
COPI: Coatomer complex I
COPs: Coat proteins
COPA: Coatomer protein complex subunit alpha
OS: Overall survival
ES: Enrichment score
NES: Normalized enrichment score
CCK-8: Cell counting kit-8
EMT: Epithelial-mesenchymal transition.

## Supplementary Materials

Supplementary Materials Table S1: the HCC patients' basic information of TCGA cohort. Table S2: the HCC patients' basic information of ICGC cohort. Table S3: basic characteristics of six HCC cohorts from GEO database. Figure S1: COPB2 expression analysis of four HCC cohorts from GEO database.
