# Supplementary material for: COPB2: A Novel Prognostic Biomarker That Affects Progression of HCC
Source: Biomed Res Int. 2021 Mar 20;2021:6648078. doi: 10.1155/2021/6648078 (PMC8007342; doi:10.1155/2021/6648078)
Supplement: Supplementary Materials — Table S1: the HCC patients' basic information of TCGA cohort. Table S2: the HCC patients' basic information of ICGC cohort. Table S3: basic characteristics of six HCC cohorts from GEO database. Figure S1: COPB2 expression analysis of four HCC cohorts from GEO database. [file 6648078.f1.docx]

**Tables：**

**Table S1.** TCGA-LIHC patient characteristics

| Clinical characteristics | | Total (370) | % |
| --- | --- | --- | --- |
| Age | ≤60 | 177 | 47.838 |
|  | ＞60 | 193 | 52.162 |
| Gender | Female | 121 | 32.703 |
|  | Male | 249 | 67.297 |
| T stage | T1 | 181 | 48.919 |
|  | T2 | 93 | 25.135 |
|  | T3 | 80 | 21.622 |
|  | T4 | 13 | 3.514 |
|  | Tx | 1 | 0.270 |
|  | Missing | 2 | 0.541 |
| M | M0 | 266 | 71.892 |
|  | M1 | 4 | 1.081 |
|  | Mx | 100 | 27.027 |
|  | Missing | 0 | 0 |
| N | N0 | 252 | 68.108 |
|  | N1 | 4 | 1.081 |
|  | Nx | 113 | 30.541 |
|  | Missing | 1 | 0.270 |
| Stage | Ⅰ | 171 | 46.216 |
|  | Ⅱ | 85 | 22.973 |
|  | Ⅲ | 85 | 22.973 |
|  | Ⅳ | 5 | 1.351 |
|  | Missing | 24 | 6.486 |
| Grade | G1 | 55 | 14.865 |
|  | G2 | 177 | 47.838 |
|  | G3 | 121 | 32.703 |
|  | G4 | 12 | 3.243 |
|  | Missing | 5 | 1.351 |
| Status | Alive | 240 | 64.865 |
|  | Dead | 130 | 35.135 |

**Table S2.** ICGC HCC patient characteristics (project: LIRI-JP)

| Clinical characteristics | | Total (232) | % |
| --- | --- | --- | --- |
| Age | ≤60 | 50 | 21.55172 |
|  | ＞60 | 182 | 78.44828 |
| Gender | Female | 61 | 26.2931 |
|  | Male | 171 | 73.7069 |
| Stage | Ⅰ | 36 | 15.51724 |
|  | Ⅱ | 106 | 45.68966 |
|  | Ⅲ | 71 | 30.60345 |
|  | Ⅳ | 19 | 8.189655 |
|  | Missing | 0 | 0 |
| Grade | G1 | 32 | 13.7931 |
|  | G2 | 121 | 52.15517 |
|  | G3 | 58 | 25 |
|  | G4 | 1 | 0.431034 |
|  | Missing | 20 | 8.62069 |
| Status | Alive | 189 | 81.46552 |
|  | Dead | 43 | 18.53448 |

**Table S3.** Basic characteristics of 6 HCC cohort from GEO database

| Cohort ID | Publication years | Platform | Country | Number of samples | | | |
| --- | --- | --- | --- | --- | --- | --- | --- |
|  |  |  |  | Nontumor | Tumor | N/T pairs | Total |
| GSE76427 | 2017 | GPL10558 | Singapore | 52 | 115 | 52 | 167 |
| GSE14520 | 2010 | GPL3921 | USA | 220 | 225 | 214 | 445 |
| GSE39791 | 2014 | GPL10558 | USA | 72 | 72 | 72 | 144 |
| GSE36411 | 2012 | GPL10558 | South Korea | 42 | 42 | Not available | 84 |
| GSE102079 | 2018 | GPL570 | Japan | 105 | 152 | Not available | 257 |
| GSE25097 | 2011 | GPL10687 | USA | 289 | 268 | Not available | 557 |

**Figure:**


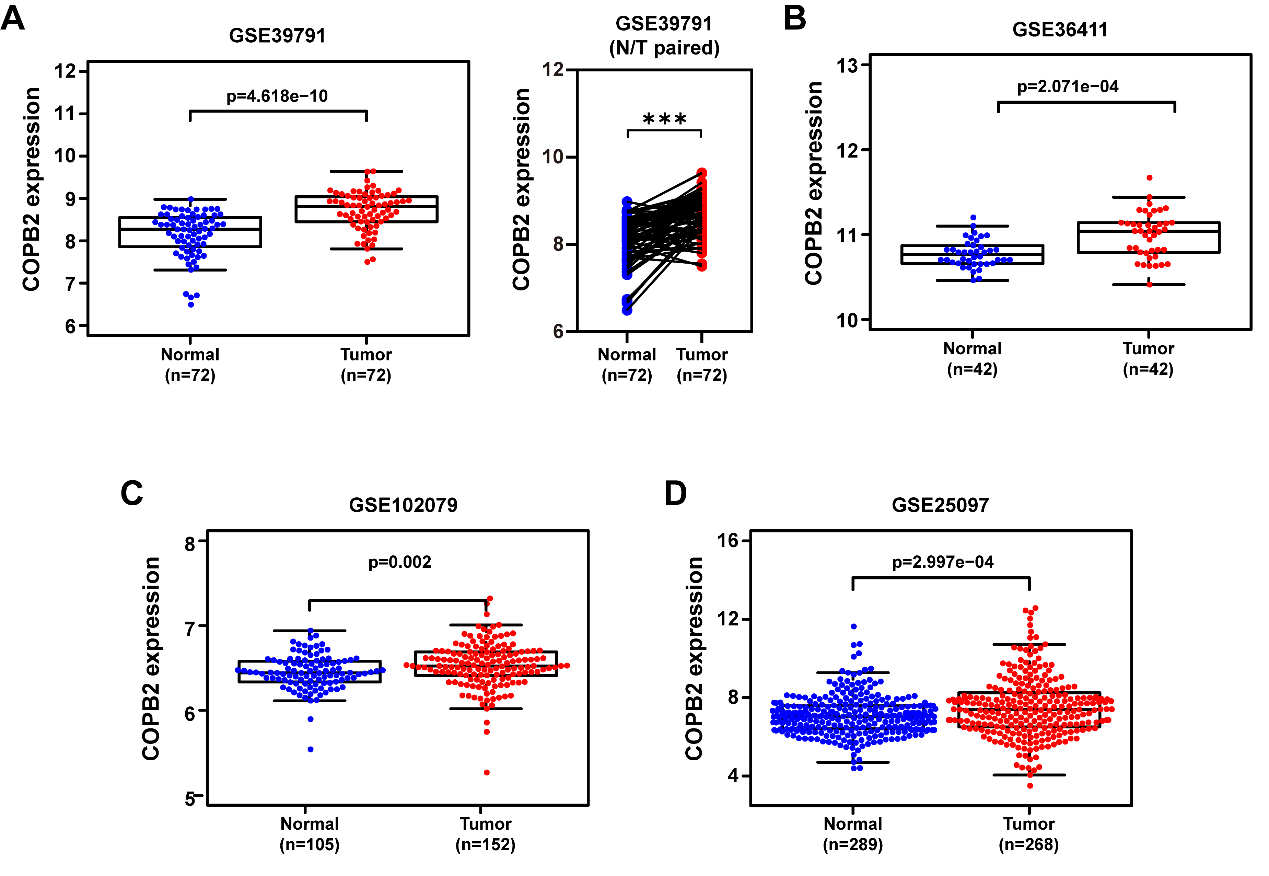


**Figure S1: COPB2 expression analysis of 4 HCC cohort from GEO database.**
